# Dataset for surface and internal damage after impact on CFRP laminates

**DOI:** 10.1016/j.dib.2022.108462

**Published:** 2022-07-10

**Authors:** Saki Hasebe, Ryo Higuchi, Tomohiro Yokozeki, Shin-ichi Takeda

**Affiliations:** a Department of Aeronautics and Astronautics, The University of Tokyo, 7-3-1 Hongo, Bunkyo-ku, Tokyo 113-8656 Japan; b Aeronautical Technology Directorate, Japan Aerospace Exploration Agency (JAXA), 6-13-1 Osawa, Mitaka-shi, Tokyo 181-0015, Japan

**Keywords:** BVID, Machine learning, Thermoset CFRP, Non-destructive testing

## Abstract

Various foreign objects can collide with CFRP structures, such as CFRP aircraft. Once something impacts with CFRP laminates, both surface damage and internal damage can occur. Even if the external damage is such invisible as called barely visible impact damage, there are matrix cracks or delamination that are the main cause of compressive strength reduction, so it is difficult to find the relationship between external and internal damage on CFRP laminates. This dataset is prepared for predicting impact information only from surface damage profiles using Machine Learning (Hasebe et al., 2022). It includes three data, surface damage image (png), surface depth contour image(png), and internal damage image after ultrasound C-scanning (jpg) after low-velocity impact testing under various impact conditions. The data are helpful for researchers and engineers who deal with the impact behavior of CFRP or data science.

**Specifications Table**

**Table:** 

Subject	Engineering
Specific subject area	Impact damage on composite materials, and Machine learning
Type of data	Table Image
How the data were acquired	1. Drop weight impact testing machine CEAST 9350 (Instron Corporation)
	2. 3D surface profiler VR-5000 and its software, VR-5000 series application (Keyence Corporation) for png data
	3. Ultrasound C-scanning system HIS3 LF (KJTD Co., Ltd.) for jpg data
Data format	Raw Analyzed
Description of data collection	Data were collected under various impact conditions. Details are described in this article.
Data source location	Data were obtained from the Aoki-Yokozeki lab, department of Aeronautics and Astronautics, The University of Tokyo, Japan, and Aeronautical Technology Directorate, Japan Aerospace Exploration Agency, Japan.
Data accessibility	With the article
	Repository name: Mendeley Data
	Data identification number:
	10.17632/74t7kcdgkr.1
	10.17632/yfxyg8jm46.1
	10.17632/xcmzfsbd9t.1
	10.17632/ykhs7s2dck.1
	10.17632/w68dtmpfyf.1
	10.17632/cgtnjyggtm.1
	10.17632/6zt73pcnxv.1
	Direct URL to data:
	https://data.mendeley.com/datasets/74t7kcdgkr/1
	https://data.mendeley.com/datasets/yfxyg8jm46/1
	https://data.mendeley.com/datasets/xcmzfsbd9t/1
	https://data.mendeley.com/datasets/ykhs7s2dck/1
	https://data.mendeley.com/datasets/w68dtmpfyf/1
	https://data.mendeley.com/datasets/cgtnjyggtm/1
	https://data.mendeley.com/datasets/6zt73pcnxv/1
Related research article	S. Hasebe, R. Higuchi, T. Yokozeki, S. Takeda, Internal low-velocity impact damage prediction in CFRP laminates using surface profiles and machine learning, Composites Part B: Engineering, Volume 237, 2022. doi:10.1016/j.compositesb.2022.109844.

**Value of the Data**

• The data can be used by researchers, engineers, and designers who handle CFRP structures.
• Some complicated machine learning algorithms can be constructed with this dataset.
• This data is helpful to investigate the difference of impact damage depending on each impact condition.
• To our best knowledge, this is the first dataset on CFRP impact damage under various impact conditions publically available.

## 1 Data Description

**Fig. 1:**
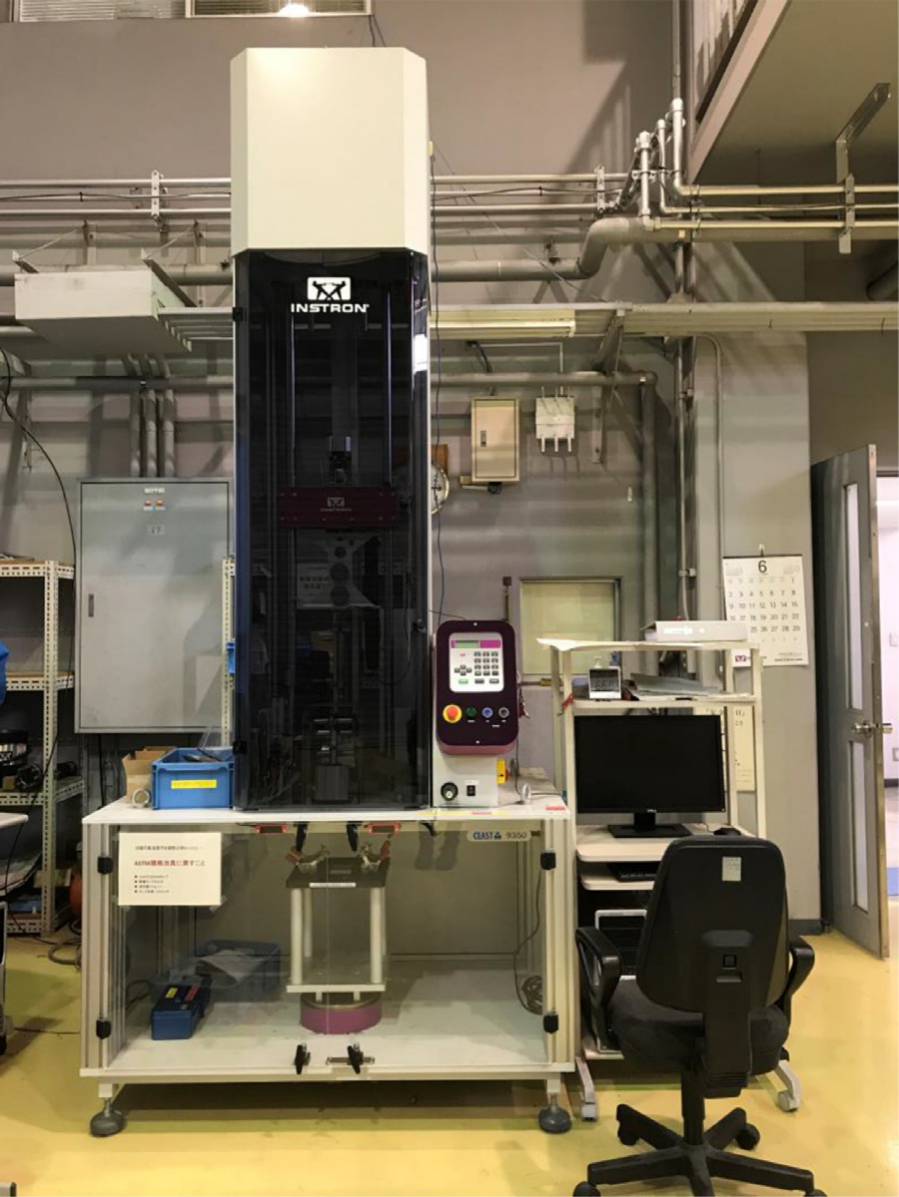
Drop tower impact system CEAST 9350.

**Fig. 2:**
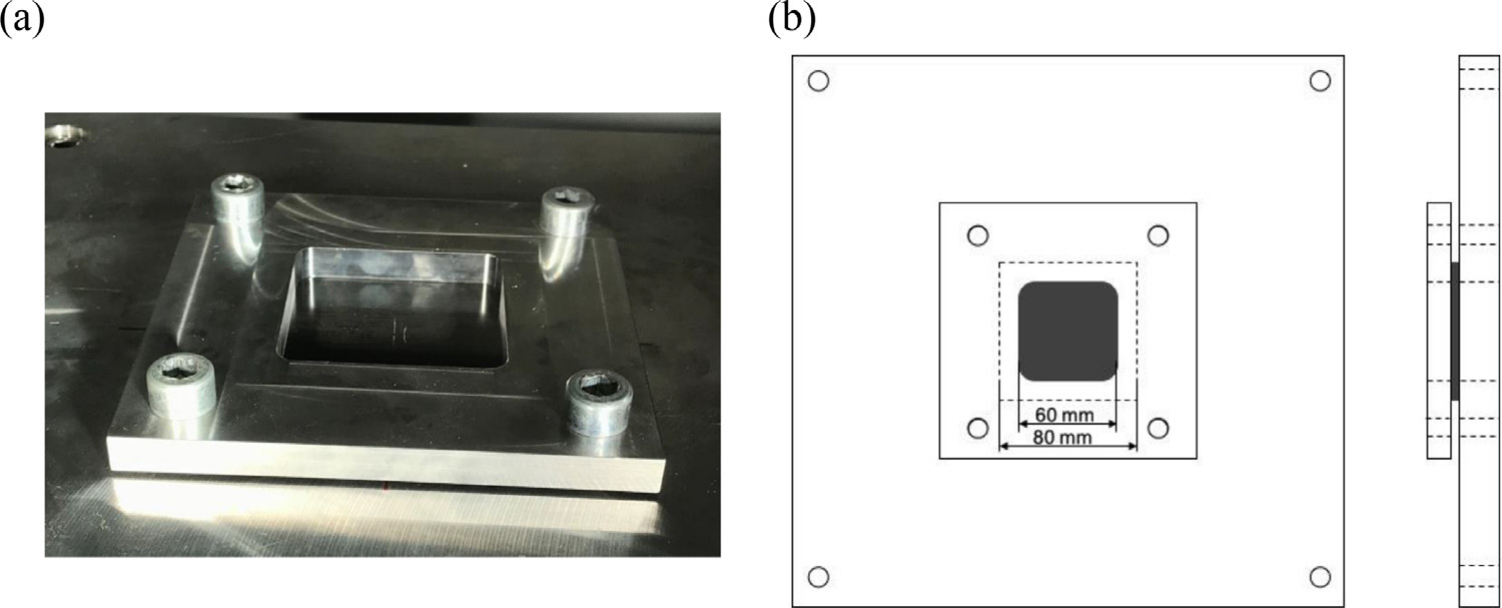
How to fix the specimens : (a) image and (b) diagram.

**Fig. 3:**
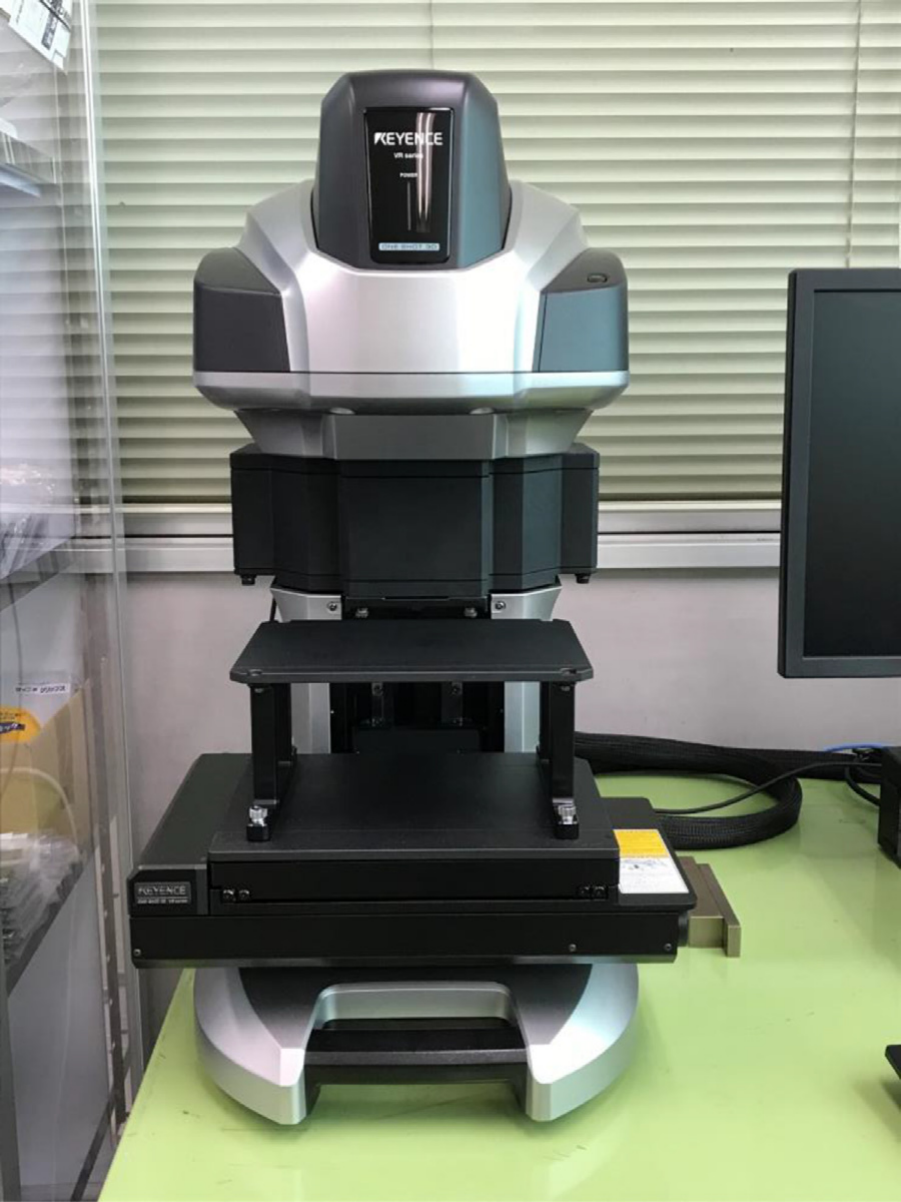
Wide-area 3D measurement system VR-5000.

**Fig. 4:**
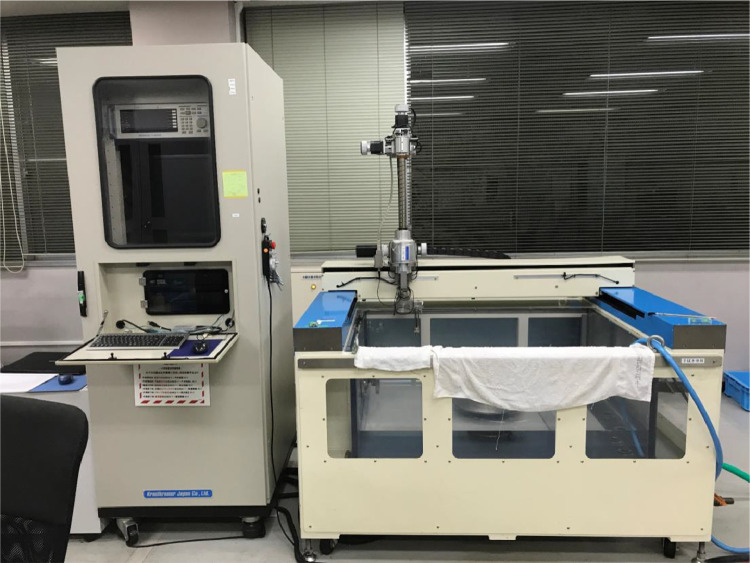
Ultrasound C-scanning system HIS3.

**Fig. 5:**
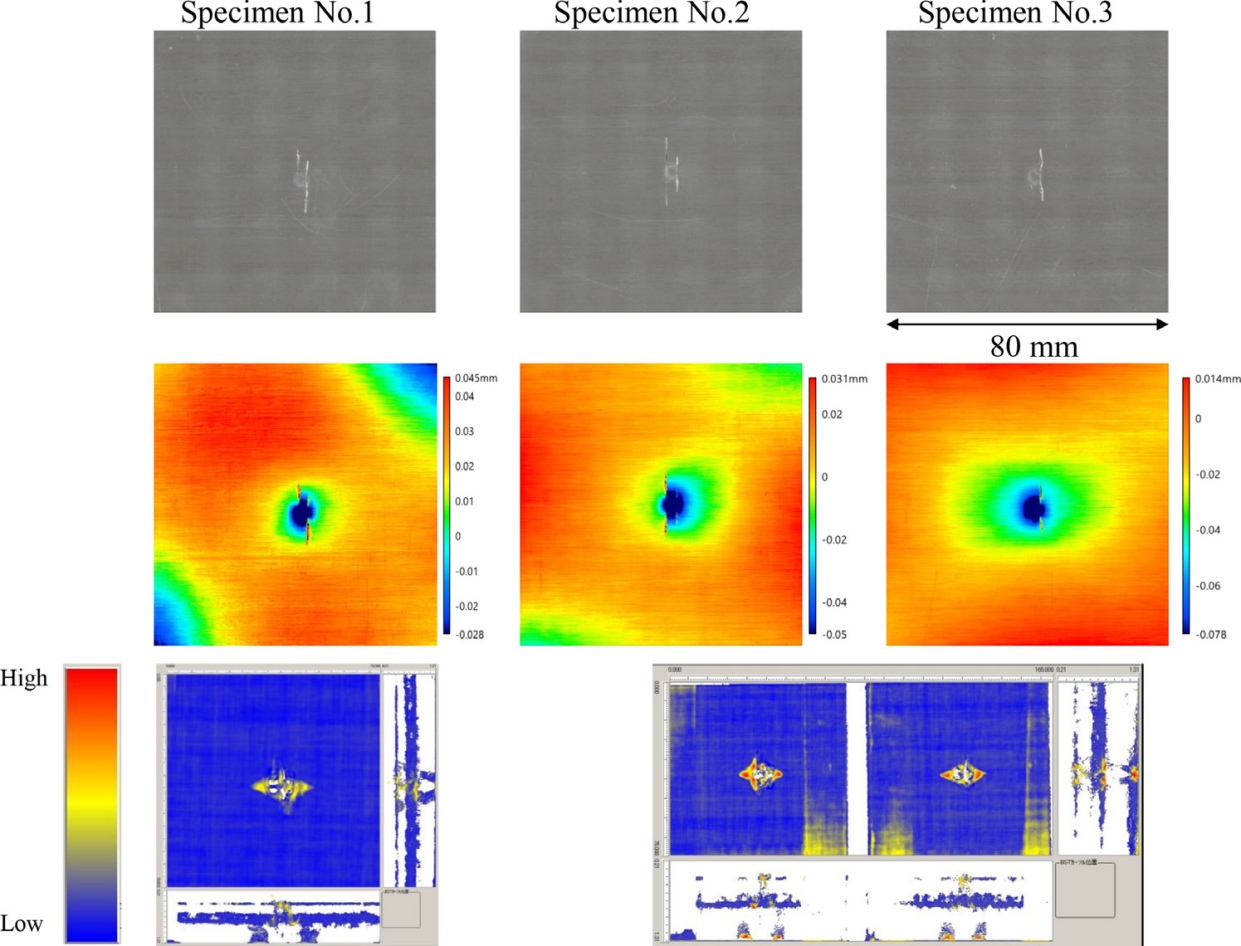
(Top to bottom) Surface images, depth contour plot, and C-scanning of C8/HemiA.

**Fig. 6:**
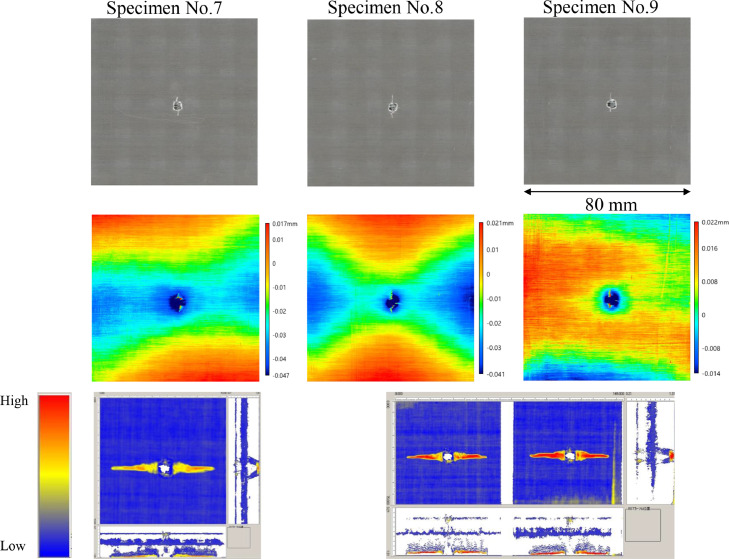
(Top to bottom) Surface images, depth contour plot, and C-scanning of C8/Coni60.

**Fig. 7:**
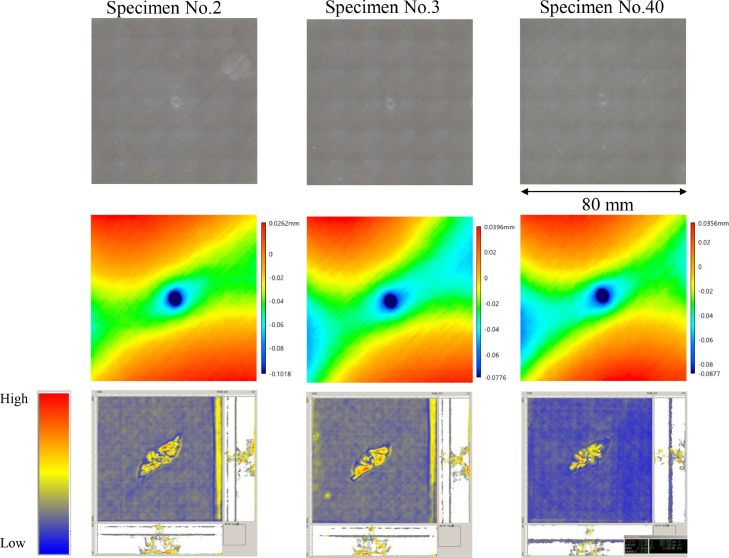
(Top to bottom) Surface images, depth contour plot, and C-scanning of Q8/HemiA.

**Fig. 8:**
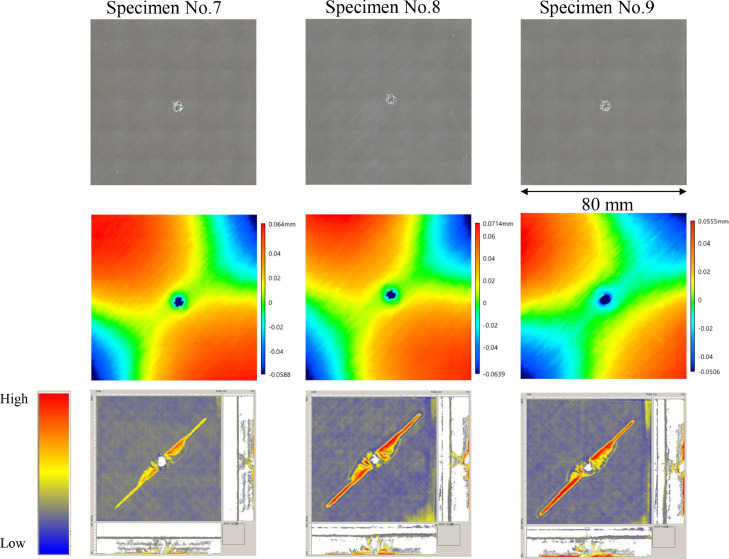
(Top to bottom) Surface images, depth contour plot, and C-scanning of Q8/Coni60.

**Fig. 9:**
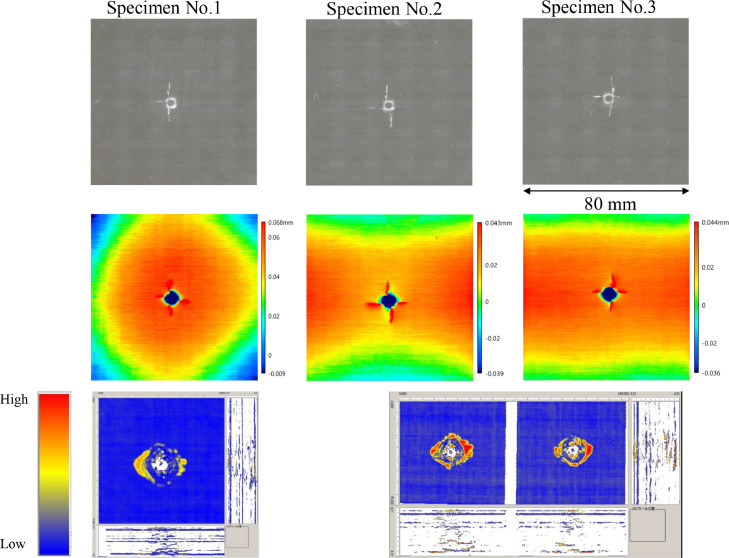
(Top to bottom) Surface images, depth contour plot, and C-scanning of C24/HemiA.

**Fig. 10:**
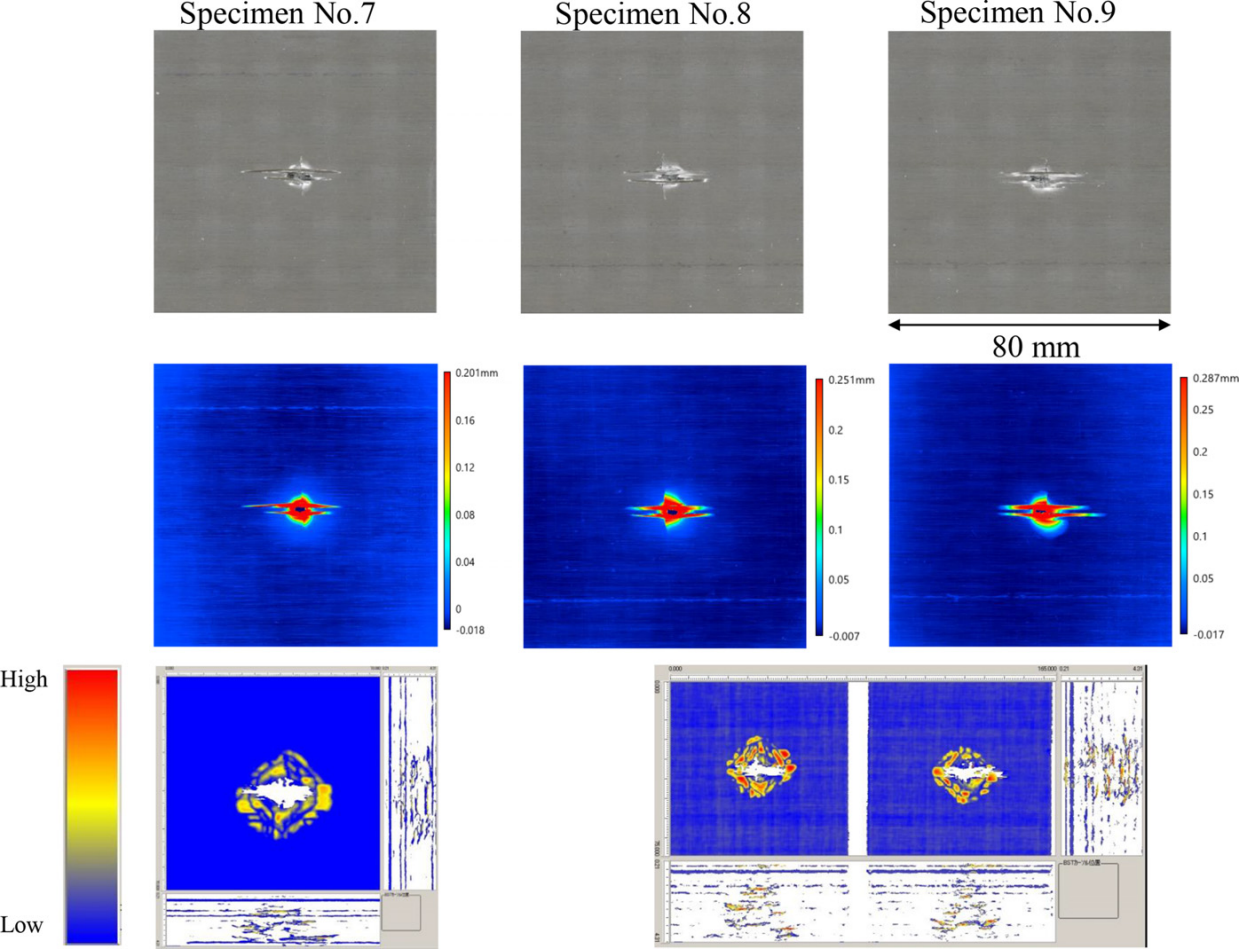
(Top to bottom) Surface images, depth contour plot, and C-scanning of C24/Coni60.

**Fig. 11:**
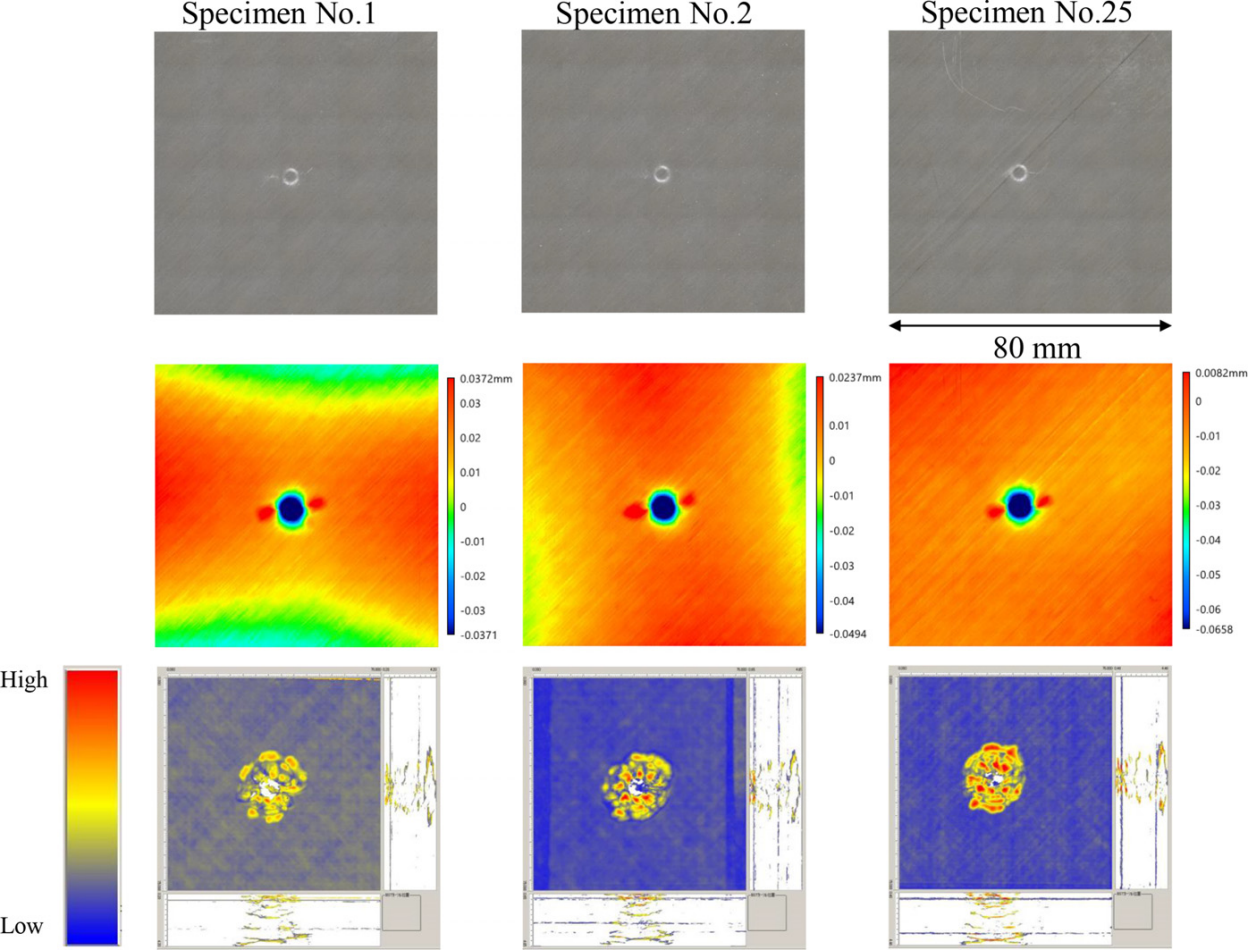
(Top to bottom) Surface images, depth contour plot, and C-scanning of Q24/HemiA.

**Fig. 12:**
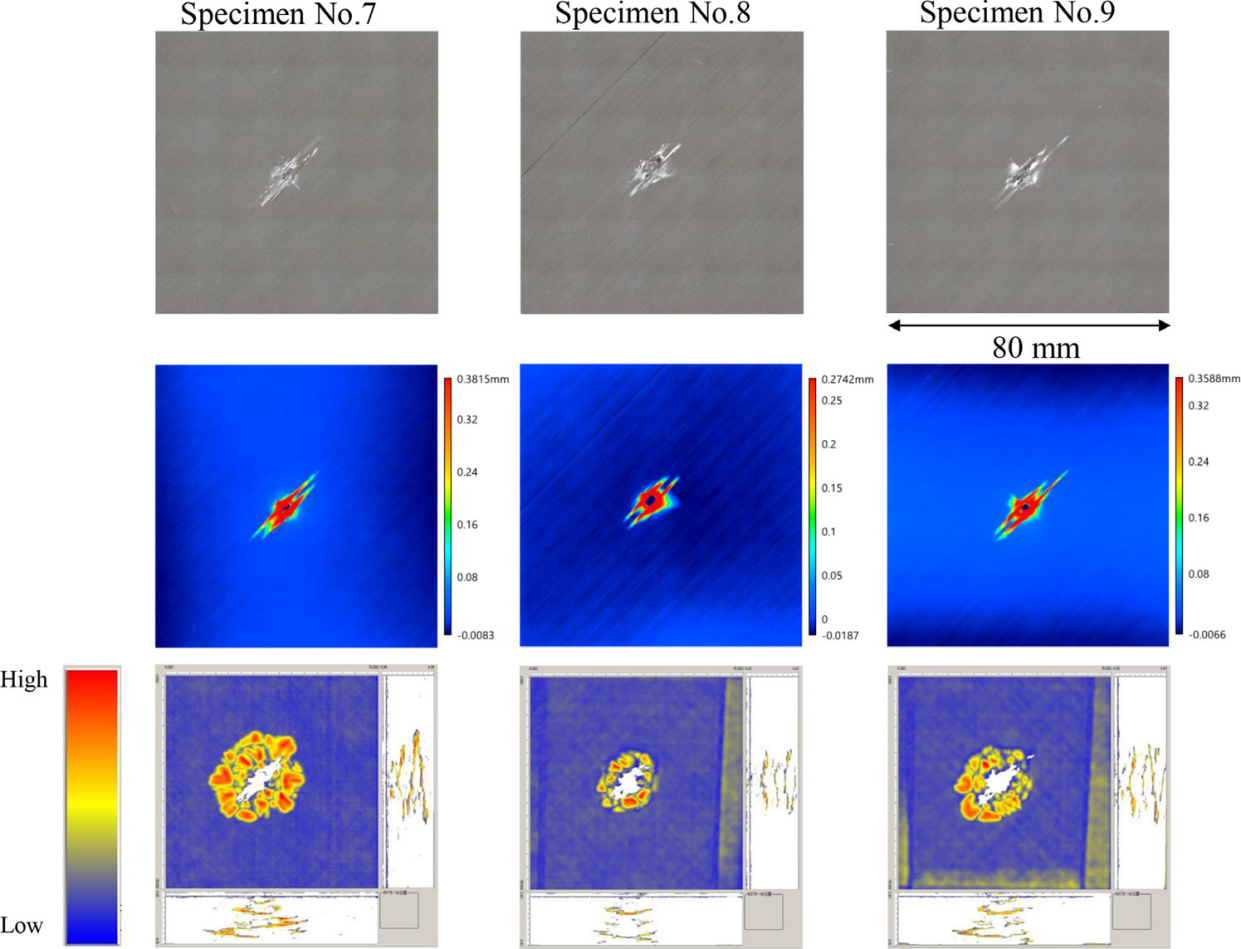
(Top to bottom) Surface images, depth contour plot, and C-scanning of Q24/Coni60.

The data consists of the external damage image, surface depth contour plot and internal damage image of carbon fiber reinforced plastics (CFRP) after low-velocity impact (LVI) testing. The impact test was carried out using the Drop Tower Impact System CEAST 9350 (Instron Corporation) under various impact conditions (Fig. 1, Table 1). The specimens were put between two plates to fix (Fig. 2). Three specimens were prepared for each impact condition. After the LVI test, a wide area measurement system VR-5000 (Keyence Corporation) and the ultrasound C-scanning system HIS3 (KJTD Co., Ltd.) were utilized to measure external damage (Figs. 3 and 4). Fig. 5, Fig. 6, Fig. 7, Fig. 8, Fig. 9, Fig. 10, Fig. 11, Fig. 12 describe (a) Sample surface image, (b) depth contour plot, and (c) B scope and C scope as described in Table 1.

**Table 1:** Impact conditions.

Specimen No.	Laminates	Impactors	Impact energy [J/mm]	Figure No.
1, 2, 3	C8 ([0/90]${}_{\text{2s}}$)	HemiA	3.35	5
7, 8, 9	C8([0/90]${}_{\text{2s}}$)	Coni60	3.35	6
2, 3, 40	Q8([45/0/-45/90]${}_{\text{s}}$)	HemiA	3.35	7
7, 8, 9	Q8([45/0/-45/90]${}_{\text{s}}$)	Coni60	3.35	8
1, 2, 3	C24([0/90]${}_{\text{6s}}$)	HemiA	3.35	9
7, 8, 9	C24([0/90]${}_{\text{6s}}$)	Coni60	3.35	10
1, 2, 25	Q24([45/0/-45/90]${}_{\text{3s}}$)	HemiA	3.35	11
7, 8, 9	Q24([45/0/-45/90]${}_{\text{3s}}$)	Coni60	3.35	12

All dataset can be found in Mendeley Data[2], [3], [4], [5], [6], [7], [8], each separated by layups and specimen size (C8, C16, C24, Q8, Q16, Q24, and ASTM specimens). Each Mendeley data consists of

1. ”Impacted surface image” folder
2. ”Internal damage” folder
3. ”Non-impacted surface image” folder
4. ”Surface profile” folder
5. ”Impact condition” file

”Impacted surface image” folder contains the images off impacted surface, which are the outputs of VR-5000. In ”Internal damage” folder, the screenshots of the c-scanning result are stored. In ”non-impacted surface image” folder, the opposite surface images of those in ”impacted surface image” folder are saved. Data in ”Surface profile” folder is the out-of-plane displacement of impacted surface before and after the impact test. It is able to duplicate the depth contour plots in Fig. 5, Fig. 6, Fig. 7, Fig. 8, Fig. 9, Fig. 10, Fig. 11, Fig. 12 using these data. Also, all data in the related paper[1] is based on these surface profile data. ”Impact condition.xlsx” explains each impact condition of the Mendeley data.

## 2 Experimental Design, Materials and Methods

### 2.1 Material and Specimens

This research experimented with T800S/3900-2B, an interface toughened material manufactured by Toray Industries Inc. Although the material properties are not evalated in this study, the previous research conducted various experiments to collect them [9]. The size of the specimen was cut into 80mm×80mm from large plates.

### 2.2 Low-Velocity Impact Test

The testing machine, CEAST 9350 (Fig. 1) confirms to ASTM D7136[10], and the followings were arranged for this experiment [1]:

• The specimen size (see 1)
• The fixing tools (see Fig. 2)
• The impactor shapes
• The impact energy : The energy is calculated using the specimen thickness. Since the mass of the impactor is known, the initial height is obtained.

To reproduce these data, put the specimen on the center of fixing tools. Otherwise, the boundary condition lacks uniformity. Table 1 lists the impact conditions (the stacking sequence, the impactor shape and the impact energy) of the data which is mentioned in this paper. The ply thickness is about 0.1875 mm.

### 2.3 Surface Damage Measurement

The software attached to VR-5000 (Fig. 3) was used to analyze the raw surface profile data whose extension is unique to them (.zon). As the first step of the analysis, it is necessary to define a reference plane (i.e., undeformed intact surface) based on the mean-square fitting from four corners of each specimen. Here, the relative positions of four corners are considered to be consistent before and after the LVI test since all of the four edges were fixed during the test. After defining the reference plane, the software calculates each depth on the specimen. Then, it is able to obtain each depth contour plot in Fig. 5, Fig. 6, Fig. 7, Fig. 8, Fig. 9, Fig. 10, Fig. 11, Fig. 12. This software considers the bulge direction to be positive.Each impact condition is summarized in Table 1.

### 2.4 Internal Damage Measurement

In order to evaluate the internal damage of the impacted specimen, ultrasound C-scanning was conducted (Fig. 4). Table 2 lists the measurement configuration of C-scanning. As the software cannot export raw file in a non-proprietary format, the screenshot was taken as image data. In Fig. 5, Fig. 6, Fig. 7, Fig. 8, Fig. 9, Fig. 10, Fig. 11, Fig. 12, the C-scanning data of the same impact damage are also shown. The color represents the amplitude level.

**Table 2:** Ultrasound c-scanning measurement conditions.

Parameter	Value
Probe	3.5 MHz
Scanning pitch	0.200 mm x 0.200 mm
Scanning length	75.000 mm x 75.000 mm

## CRediT authorship contribution statement

**Saki Hasebe:** Methodology, Investigation, Writing – original draft. **Ryo Higuchi:** Conceptualization, Writing – review & editing. **Tomohiro Yokozeki:** Supervision. **Shin-ichi Takeda:** Validation, Supervision.

## Declaration of Competing Interest

The authors declare that they have no known competing financial interests or personal relationships that could have appeared to influence the work reported in this paper.

## Data Availability

Datasets on CFRP specimens after the ASTM standard low-velocity impact tests (Original data) (Mendeley Data). Datasets on CFRP specimens after low-velocity impact tests: 24-layer quasi-isotropic laminates (Original data) (Mendeley Data). Datasets on CFRP specimens after low-velocity impact tests: 16-layer quasi-isotropic laminates (Original data) (Mendeley Data). Datasets on CFRP specimens after low-velocity impact tests: 8-layer quasi-isotropic laminates (Original data) (Mendeley Data). Datasets on CFRP specimens after low-velocity impact tests: 24-layer cross-ply laminates (Original data) (Mendeley Data). Datasets on CFRP specimens after low-velocity impact tests: 16-layer cross-ply laminates (Original data) (Mendeley Data). Datasets on CFRP specimens after low-velocity impact tests: 8-layer cross-ply laminates (Original data) (Mendeley Data).
